# A transaminase with β-activity from *Variovorax boronicumulans* for the production of enantiopure β-amino acids

**DOI:** 10.1016/j.heliyon.2022.e12729

**Published:** 2022-12-30

**Authors:** Uwe Wegner, Falko Matthes, Nicolaus von Wirén, Mohammad-Reza Hajirezaei, Rüdiger Bode, Gotthard Kunze, Marion Rauter

**Affiliations:** a Leibniz Institute of Plant Genetics and Crop Plant Research (IPK), Corrensstr. 3, OT Gatersleben, D-06466 Seeland, Germany; b Orgentis Chemicals GmbH, Bahnhofstr. 3-5, OT Gatersleben, D-06466 Seeland, Germany; c Institute of Microbiology, University of Greifswald, Felix-Hausdorff-Str. 8, D-17489 Greifswald, Germany

**Keywords:** ω-transaminase, β-Amino acid, *Variovorax boronicumulans,* stereo-selectivity, Kinetic resolution

## Abstract

Enantioselective transamination of amino acids is a great challenge in biotechnology as suitable enzymes with wide substrate spectrum are rare. Here, we present a new transaminase from *Variovorax boronicumulans* (VboTA, *Variovorax boronicumulans*ω-transaminase) which is specific for β-amino acids.

The amino acid sequence of VboTA is similar to an ω-transaminase from *Variovorax paradoxus*, for which a crystal-structure is available. This similarity is allowing us to classify VboTA as a fold type 1 ω-transaminase (ω-TA). Although both enzymes have a high sequence similarity (86% identities, 92% positives), there are differences in the active center, which allow VboTA to accept a broader substrate spectrum. Both enzymes have also a different temperature stability and temperature optimum.

VboTA deaminates the D-form of aromatic β-amino acids, such as β-homophenylalanine and β-phenylalanine as well as aliphatic β-amino acids, such as β-homoalanine and β-leucine. The optimal reaction conditions turned out to be 32 °C and pH 9. Kinetic resolution lead to high enantiomeric excess of 86.6% to >99.9%, depending on the amino donor/acceptor pair. In contrast to many other ω-TAs, VboTA has a broad substrate spectrum and uses both aromatic or aliphatic amino acids. With γ-amino acids as substrates, VboTA showed no activity at all.

## 1 Introduction

Enantiopure β- and γ-amino acids are interesting compounds for the pharmaceutical industry [1,2]. They are used in curing various neurological diseases like epilepsy or anxiety disorders [[3], [4], [5], [6], [7]] or to treat cancer [8,9]. They are important precursors for a huge variety of heterocyclic compounds [10] or can be applied in the production of therapeutical peptides [11]. These peptides fold into a similar shape as peptides consisting of their α-amino acid homologues [[12], [13], [14], [15], [16]], but they exhibit a higher resistance against proteolytic degradation [[17], [18], [19], [20]].

Their efficient synthesis in optically pure form is a great challenge. Chemical synthesis requires cost-intensive enantiospecific catalysts [21] or racemic mixtures [22,23] have to be separated in a complex process. This is why the application of biocatalytic strategies attracted attention over the last two decades [24].

ω-Transaminases (ω-TAs) got into focus for the synthesis of β- and γ-amino acids due to their high versatility of transformable substrates [25]. ω-TAs are PLP (pyridoxal phosphate)-dependent enzymes, which carry the transferred amino group just transiently during a reaction cycle [26] without any co-factor that needs to be regenerated [27].

There are two general reaction modes for the production of β- or γ-amino acids using ω-TAs: the kinetic resolution or asymmetric synthesis.

In the kinetic resolution an amino-group of one enantiomer is transferred to an amino acceptor, leaving the other enantiomer unaltered. The degradation of one enantiomer is the reason why a maximum yield of only 50% can be achieved. The ω-TA catalyzed asymmetric synthesis starts with a β- or γ-keto acid as non-chiral precursor, which is selectively aminated with the help of an amino donor. Yields up to 100% are theoretically possible, but the reaction equilibrium lies on the side of the reactants. Another problem is that the substrates for the synthesis of β-amino acids, β-keto acids, decarboxylate spontaneously [28,29]. To circumvent this limitation, esters of β-keto acids are used as substrates. However, an esterase is necessary to hydrolyze the ester before the β-keto acid can be aminated [30].

ω-TAs are already in use for the production of optically pure amines. For example, a modified ω-TA from *Arthrobacter* sp. catalyses the amination of the precursor molecule of sitagliptin [31], a medication against diabetes mellitus type 2. Several mutants of an ω-TA from *Ochrobactrum anthropi* were generated by Shin et al. (2019) to increase the enzyme's activity against ketones and thus improve the efficiency of chiral amine production [32]. There are also attempts to produce γ-amino acids with ω-TAs, using enzymes from *Polaromonas* sp. and *Burkholderia graminis*. Both enzymes were applied for kinetic resolution as well as asymmetric synthesis with an enantioselectivity of more than 99% and a broad substrate spectrum [33].

Nevertheless, the availability of enzymes with a sufficiently large substrate spectrum for the production of β- and γ-amino acids is still relatively low, which emphasizes the need for identification of new potent ω-TAs.

In this study, we describe a novel ω-TA from *V. boronicumulans* which can be used for the synthesis of aromatic and aliphatic β-amino acids.

*In-silico* analysis with NCBI's prokaryotic genome annotation pipeline (PGAP) [34,35] revealed that the gram-negative soil bacterium *V. boronicumulans* [36] has a large number of transaminases, among them eight potential ω-TAs [37] with high sequence homology. These can roughly be distinguished according to their peptide length. Members of the first group have 433 amino acids (aa), while members of the other group have 463 aa, with proteins of both groups showing a high sequence homology of ≥94%. Here, one of the 433 aa-sized proteins is described. Its sequence is similar (86% identities, 96% positives) to an ω-TA of *V. paradoxus* (structure available on RCSB [38], entry: 4AOA [39]) and it folds *in silico* [40] to the same structure. Although there are differences in the active center, we suppose that the enzyme can be classified as a fold-type 1 transaminase [41].

## 2 Material and methods

### 2.1 Bacterial strains

*V. boronicumulans* (DSM No. 21722) was ordered from DSMZ (German Collection of Microorganisms and Cell Cultures GmbH, Braunschweig, Germany), *E. coli* strains BL21 (DE3) and XL1-Blue [*recA1 endA1 gyrA96 thi-1 hsdR17 supE44 relA1 lac* [F' *proAB lacI*^*q*^*ZΔM15* Tn*10* (Tet^r^)]] were ordered from Stratagene (CA, US).

### 2.2 Sequence alignment between different ω-TAs

We analyzed the similarities and differences of two groups of ω-TAs. One group contained enzymes which accept only aliphatic substrates. The other group contained ω-TAs which accept aliphatic as well as aromatic substrates. Sequence alignments were performed for each group and one for all enzymes. This revealed the differences and similarities between these groups of ω-TAs. As examples for the first kind of transaminases the sequences of enzymes from *Achromobacter denitrificans* (NCBI accession AAP92672.1), *Chromobacterium violaceum* (NCBI accession WP_011135573.1) and *Vibrio fluvialis* (RCSB accession 5ZTX) were used [42]. For the other group sequences from *Sphaerobacter thermophilus* (1) [43], *Mesorhizobium* sp. LUK (2) [39], *V. boronicumulans* (3) [this work] and *Polaromonas* sp. JS666 (4) [44] (NCBI accessions WP_012871332.1 (1), ABL74379.1 (2), WP_095950167.1 (3) and WP_041388512.1 (4)) were chosen. The alignments were done by COBALT [45].

### 2.3 Cultivation and preparation of crude extract of *V. boronicumulans* for TA-activity assay

*V. boronicumulans* was cultivated in 50 ml trypticase soy broth (DSMZ medium 535) at 30 °C. Cells were harvested in 50 ml tubes. After centrifugation (8.500×*g*, 15 min), cells were taken up in 1 ml TE-buffer (10 mM Tris-HCl, 1 mM EDTA, pH 8.0, Tris: Merck, Darmstadt, Germany, HCl + EDTA: Roth, Karlsruhe, Germany).

200 μl silica beads were added to the cell suspension. Cells were disrupted by using a pebble mill (MM 400, Retsch GmbH, Haan, Germany) at 30 Hz for 3 min. Silica beads and cell debris were removed by centrifugation at 16,000×*g* for 5 min. The supernatant was tested for ω-TA activity using a colorimetric assay, based on the quantification of α-keto acids (see 2.7).

### 2.4 Construction of *vbota* expression plasmids and generation of an *E. coli* production strain

The gene representing the best hit of the BLAST homology search (*vbota*) was optimized for *E. coli* codon usage using an online tool [46] and synthesized by Eurofins (Eurofins, Luxemburg). The gene was delivered in a pEX-vector. The vector was used to transform *E. coli* XL1-Blue cells according to the provider's manual. Transformed cells were grown overnight in 3 ml LB (Sigma, Darmstadt, Germany), containing 50 μg/ml ampicillin (Applichem, Darmstadt, Germany). The gene was excised from the vector using NdeI and XhoI (Thermo Scientific, Bremen, Germany) and inserted into the pET21b (+) plasmid (Novagen, Merck, Darmstadt, Germany) behind the inducible T7 promotor. The plasmid was transformed into *E. coli* XL1-Blue cells. *E. coli* XL1 transformants were cultivated in 2 ml LB, containing 50 μg/ml ampicillin, overnight, to increase the number of plasmids. After plasmid isolation, positive constructs were used to generate production strains by transforming *E. coli* BL21 (DE3) cells with them.

### 2.5 Cultivation of the production strain

To test the production strain, 250 ml LB with 50 μg/ml ampicillin in a 1000 ml flask were inoculated with overnight pre-cultures to an OD_600 nm_ of 0.075. Afterwards the cultures were shaken at 37 °C and 180 rpm. At an OD_600 nm_ of approx. 0.8, IPTG was added to the medium to a final concentration of 1 mmol L^−1^ and the cultures were shaken for another 2 h at 37 °C and 180 rpm. To ensure that VboTA is effectively induced by IPTG, a non-induced control was also cultivated. After cultivation, cells were harvested by centrifugation (10 min, 4 °C and 9600×*g*), and disrupted in two steps. First, cells were enzymatically disrupted with lysozyme (1 mg/ml) in lysis buffer containing potassium phosphate buffer pH 8 (20 mM), NaCl (300 mM), glycerol (10% v/v), PLP (20 μM), and EDTA (1 mM). One tablet cOmplete® (Roche, Basel, Ch) was added to 12 ml buffer to protect the extract from proteolytic degradation. Cells were suspended in an appropriate volume of lysis buffer, and incubated at 4 °C for 60 min. This was followed by a sonication step (Banduin electric UW 2070), 3 × 1 min at 70% power output, with 30 s breaks between the steps. During the sonication process and the breaks, the cells were kept on ice. Cell debris were removed by centrifugation (6000×*g*, 4 °C). VboTA was purified from this extract by Ni-affinity chromatography by adding 3 ml of Ni-NTA (Abcam, Cambridge, UK) to the crude extract, followed by overnight shaking. Purification was done as described by the manufacturer. Remaining imidazole was removed with PD10 columns (GE Healthcare, Solingen, Germany). The enzyme was eluted 3 times with 3 ml PBS and stored at 4 °C until further use.

### 2.6 Measurement of protein concentrations

Concentrations of extracted proteins were determined with Roti Nanoquant®, following the manufacturer's protocol for measuring concentrations in a microtiter plate with 1:40 diluted samples. Measurements were performed with a TECAN infinite® 200 plate reader at 590 nm and 450 nm.

### 2.7 SDS-PAGE and Western-blot

Precast RunBlue™ SDS Protein Gels from Expedeon were used (10%, 12 wells) for SDS-PAGE. Staining was done with Expedeon Instant Blue®.

For immunostaining, rabbit anti-His polyclonal antibody (MicroMol 1 mg/ml, Art. 413, 1:10,000 dilution) was used. The secondary antibody was goat anti rabbit (Abcam, Art. 6722, 1:15,000 dilution). Staining was done with NBT/BCIP tablets (Roche, Basel, Ch).

### 2.8 Determination of enzyme activity

All activity assays were performed in technical replicates with the number of replicates indicated in the legends to each figure. Activity assays with pure enzyme were performed in a volume of 100 μl in 96 well microtiter plates (Corning Inc., Corninng, NY, USA). As amino acceptors pyruvate (Boehringer-Mannheim, Mannheim, Germany), 2-oxoglutarate (Reanal, Budapest, Hungary) and oxaloacetate (Sigma, Darmstadt, Germany), each at a concentration of 2 mmol L^−1^ were used. The amino donors β-alanine, β-phenylalanine, β-homoalanine, β-homophenylalanine, β-leucine, γ-amino pentanoic acid and γ-aminobutyric acid (β-alanine was purchased from Serva, Heidelberg, Germany, all others from TCI, Tokyo, Japan) were used at a concentration of 4 mmol L^−1^. As potential amino donors, which are no amino acids, putrescine (Serva, Heidelberg, Germany), spermine and spermidine (Sigma, St: Louis, MO, USA) were used at the same concentration as the amino acids. Cofactor PLP (Sigma, St: Louis, MO, USA) was added with 0.1 mmol L^−1^ end concentration in the mixture, shortly before the reaction was started by addition of the enzyme. Two negative controls were carried out, for one approach water was added instead of enzyme, in the other approach, the amino donor was replaced by water. Plates were incubated at 30 °C for 60 min. Following, 17.5 μl of each well was transferred into a new microtiter plate. 52.5 μl water and 70 μl of 2,4-dinitrophenylhydrazine (1 mmol L^−1^, Fluka, Buchs, St. Gallen in 1 mol L^−1^ HCl) were added, which forms a hydrazone with the remaining amino donor and stops the enzymatic reaction. After 20 min at room temperature, 70 μl of 4 mol L^−1^ NaOH was added and intensively mixed. The formed hydrazonium salt can be quantified photometrically at 570 nm. The results of the negative controls were used as 100% values for the quantification of remaining amino acceptor. One Unit is defined as the conversion of 1 μmol amino acceptor per minute.

### 2.9 Biochemical characterization of VboTA

To determine the thermal stability, aliquots of VboTA were incubated at different temperatures (20–90 °C in 10 °C steps) for 60 min. Afterwards the activity of VboTA was measured with β-phenylalanine.

The optimal temperature was determined in a thermo cycler (Eppendorf Epgradient S Mastercycler). The samples were mixed in 0.5 ml tubes and incubated at temperatures from 30 to 50 °C. All other conditions were the same as for the temperature stability assay.

Inhibition of VboTA was tested with different concentrations of D/L-β-phenylalanine (20–34 mM), 2-oxoglutarate (10–18 mM), d-glutamate and l-glutamate (0–24 mM each). This assay was performed at pH 9 and at 30 °C. An inhibition by other products (e.g. acetoacetic acid) was not analyzed, because of the instability of 3-oxo-acids.

### 2.10 Determination of Km

100 μl samples with 10 μl purified VboTA in 100 mM potassium borate buffer, pH 9, 0.1 mM PLP and concentrations of 2, 4, 6, or 8 mM 2-oxoglutarate were prepared. As amino donor, β-phenylalanine (10 mM) was used. For each 2-oxoglutarate concentration four replicates were performed. For the determination of Km and Vmax for the different amino donors 5.2 mM 2-oxoglutarate were used in all samples with 2–10 mM amino donor in steps of 2 mM. The samples were incubated at 30 °C for 90 s, afterwards VboTA was inactivated at 95 °C for 5 min. All samples were stored at −20 °C until measurement. Glutamate was quantified as described in Ref. [47].

### 2.11 Kinetic resolution of β-homoalanine, β-leucine, β-homophenylalanine and β-phenylalanine by VboTA

50 mM of β-amino acids were deaminated with 50 mM sodium pyruvate or 2-oxoglutarate sodium salt as well as 0.1 mM PLP in 100 mM bicine buffer pH 9. Reactions were started by the addition of purified VboTA (56 U ml^−1^ or 180 μg/ml protein concentration) and incubated at 30 °C. After 24 h reactions were stopped by incubation at 95 °C for 5 min.

### 2.12 Chiral analysis of β-homoalanine, β-leucine, β-homophenylalanine and β-phenylalanine

To analyze the kinetic resolution of β-homoalanine, β-leucine, β-homophenylalanine and β-phenylalanine the remaining β-amino acids were derivatized by 2,3,4,6-tetra-*O*-acetyl-β-d-glucopyranosyl isothiocyanate (GITC). 25 μl 1:10 diluted sample from the 50 mmol L^−1^ kinetic resolution experiments were mixed with 25 μl 11 μl ml^−1^ triethylamine (TEA) in acetonitrile (ACN) and 50 μl 5 mg ml^−1^ GITC in ACN. After 30 min at room temperature, 10 μl 2.6 μl ml^−1^ ethanolamine in ACN was added. After 2 min centrifugation at 16,000×*g* a clear supernatant was obtained and measured by HPLC. The mixtures of enantiomeres were isocratically eluted from a Nucleodur RP18 column (4.6 × 150 mm, 10 μm) using methanol: water = 3:7 + 0.1% TFA (β-homoalanine) as well as methanol: 11.5 g L^−1^ phosphoric acid in water = 55:45 (β-leucine, β-homophenylalanine) or 1:1 (β-phenylalanine) as eluents. Detection was done at 254 nm. Quantification was done via external calibration with the enantiomeric mixtures as standards in reaction mixtures without enzymes.

## 3 Results

### 3.1 Identification of an ω-transaminase from *V. boronicumulans*

*V. boronicumulans* was cultivated and the intracellular soluble fraction (crude extract) was tested for ω-transaminase activity with β-homoalanine, β-leucine, β-phenylalanine, β-homophenylalanine and γ-amino pentanoic acid as substrates. Pyruvate was found to be aminated by *V. boronicumulans* crude extract with all 4 β-amino acids, but not with the γ-amino acid 4-amino pentanoic acid. After positive results with the crude extract, a BLAST homology search [48,49] of a known ω-TA from *Sphaerobacter thermophilus* (StoTA) [43] against the protein sequences of *V. boronicumulans* (FASTA file downloaded on February 2nd^,^ 2019 from NCBI, taxID: 436515, 57691 entries) was performed. NCBI's blastp suite was employed with default settings to identify possible ω-TAs. The best hit, with a score of 247, turned out to be the protein with the NCBI accession number WP_095950167.1 (a predicted aminotransferase class III-fold pyridoxal phosphate-dependent enzyme, hereafter referred to as VboTA). It has an identity of 36% with 53% positives compared to StoTA (Supplemental 2) and 92% positives in comparison with an ω-TA of *V. paradoxus*. Another homology search of VboTA against a putative motif for ω–transaminases [44] revealed that VboTA has all residues that are supposed to be important for the deamination of aromatic β-amino acids (Supplemental 3). Borders of secondary structures as well as conserved residues, which seem to be important for the function of aromatic β-transaminases, are identical for VboTA and the potential motif of ω-transaminases.

### 3.2 Determination of sequence similarities

All seven sequences have some amino acid residues in common. The positions refer to the sequence of VboTA. These residues are: 1 M, 57G, 65G, 81G, 90A, 120P, 128T, 129 N, 130S, 131G, 133E, 158Y, 159H, 160G, P175, 201A, 207P, 213G, 239D, 240E, 241V, 245R, 260D, 266K, 275G, 276A, 296H, 299T and 354G. A complete alignment with all identical residues can be found in supplemental 1.

**Fig. 1:**
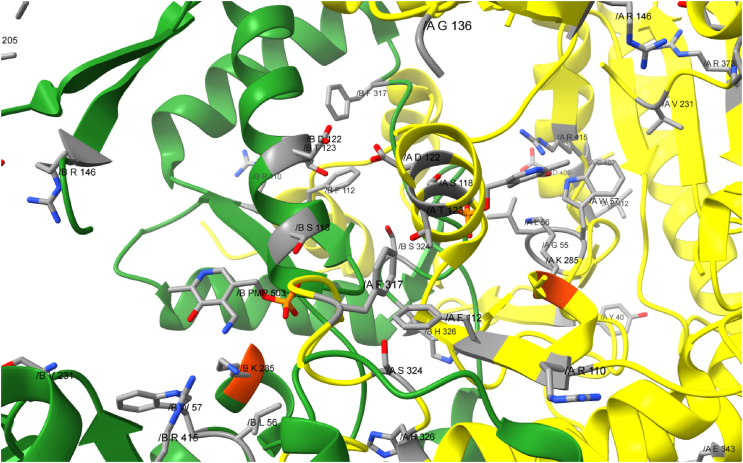
Close up of one active center of 5ZTX as a representative for aromatic ω-TAs. Yellow: chain A, green: chain B, orange: catalytic Lysine. Amino acids, which are conserved in enzymes, which accept only aliphatic amino acids, are labeled and marked in grey. These residues distinguish aliphatic ω-TAs from other ω-TAs. (For interpretation of the references to color in this figure legend, the reader is referred to the Web version of this article.)

The three enzymes which only accept aliphatic amino acids can be distinguished from the less specified transaminases by the following residues. Only amino acids, which are conserved in all three sequences of aliphatic ω-TAs, but do not appear in any of the four other sequences are considered. The positions refer to RCSB accession 5ZTX, numbers in brackets refer to the according positions in VboTA: F14 (36), T20 (37), Y40 (59), G55 (74), L56 (75), W57 (76), R110 (124), F112 (126), S118 (132), D122 (136), T123 (137), G136 (144), R146 (154), H178 (−), A202 (185), L205 (188), E206 (189), I209 (192), A214 (197), V231 (214), P234 (217), Y238 (221), G263 (244), S324 (301), H326 (303), E343 (320), R373 (351), C407 (383), D409 (385), L412 (392) R415 (395) and P426 (−). Some of these residues are part of the p-pocket or are close to it. Fig. 1 provides a closer look into one active center of 5ZTX, the conserved residues are highlighted.

**Fig. 2:**
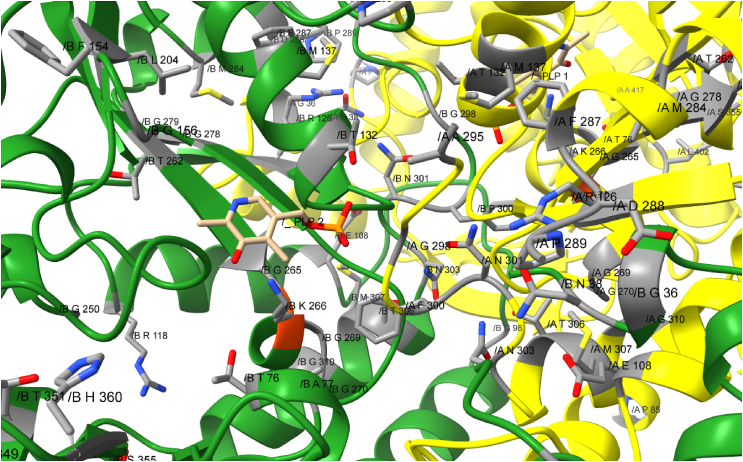
Close up of one active center of VboTA. Yellow: chain A, green: chain B, orange: catalytic lysine. Amino acids, which are conserved in enzymes, which accept aliphatic and aromatic substrates are labeled and marked in grey. (For interpretation of the references to color in this figure legend, the reader is referred to the Web version of this article.)

Several residues were conserved in the sequences of WP_012871332.1, ABL74379.1, WP_095950167.1 and WP_041388512.1, which accept both, aromatic and aliphatic amino acids. Only residues which are identical in all four sequences, but do not appear in the sequences of the aliphatic ω-TAs, are considered. These residues are (the numbers refer to VboTA): N38, P47, T76, A77, P85, G98, E108, R118, R126, T132, M137, T146, G147, F154, G156, P171, P175, N184, D185, H196, L204, F221, G250, L255, T262, G265, G269, G270, G278, G279, M284, F287, D288, P289, G298, F300, N301, N303, T306, M307, G310, L326, R334, Q349, T351, S355, H360, I392, L402 and A417. A close up to one active center of VboTA with highlighted conserved residues is given in Fig. 2.

### 3.3 Production and purification of VboTA

The *vbota* gene was expressed in *E. coli* BL21 (DE3) with a C-terminal His-tag (see Material and methods 2.4). After cultivation of the recombinant strain (see material and methods 2.5) His-tagged VboTA was purified with Ni-NTA-Agarose from the intracellular soluble fraction (crude extract).

The protein concentration decreased from 4.91 g L^−1^ to 0.31 g L^−1^ during purification. Purified VboTA was detected between the 40 and 55 kDa marker bands on a Coomassie-stained SDS-PAGE gel, as well as on the immunostained protein gel blot using an anti-His-tag specific primary antibody (Supplemental 4). This corresponds to the predicted molecular mass of approx. 47.17 kDa. Protein bands were detected primarily in the induced samples, whereas these bands were much less intensive, or missing at all, in the non-induced samples. The weaker bands, which were visible in the non-induced samples most likely result from low efficacy of the lac repressor. Thus, even without induction, small amounts of VboTA were produced.

### 3.4 Biochemical characterization

The optimum temperature and pH for ω-TA activity were investigated using purified VboTA with β-phenylalanine and β-homoalanine as substrates. The pH-optimum for both amino donors was at pH 9. Interestingly, at low pH values VboTA showed higher activity against β-phenylalanine than against β-homoalanine. While the enzyme showed more than 50% of its maximum activity against β-phenylalanine down to a pH of 5.0, optimal pH conditions were much narrower when β-homoalanine was converted, with a residual activity of less than 20% at a pH below 7.0.

**Fig. 3:**
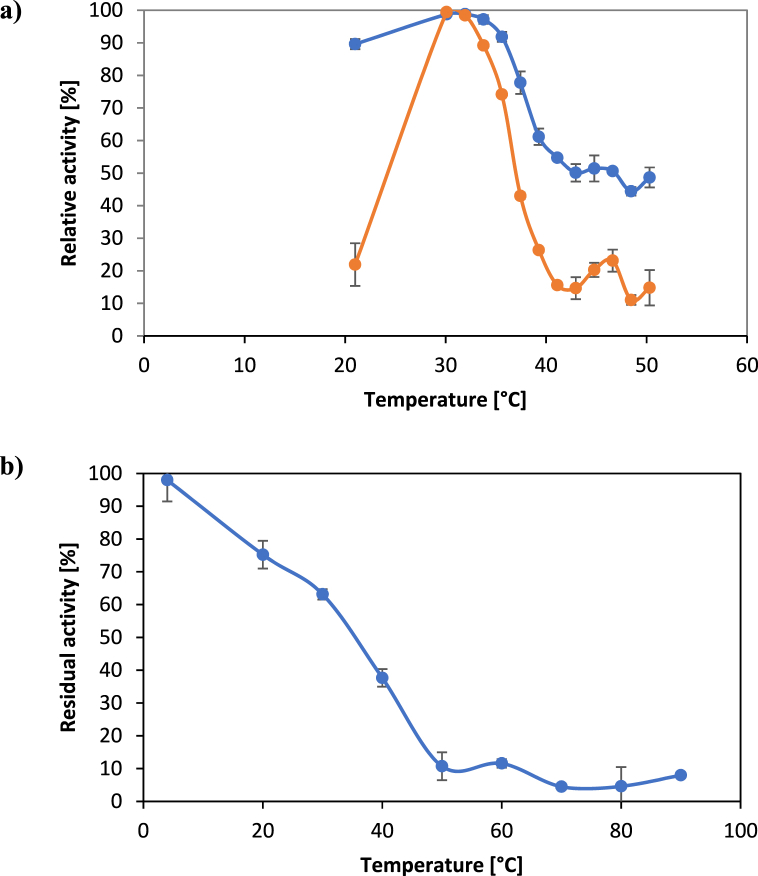
Determination of activity of VboTA under different conditions. (a) Temperature optimum. Activity of VboTA at different temperatures, dark grey: β-phenylalanine, light grey: β-homoalanine. (b) Temperature stability. Residual activity of VboTA after 1 h incubation at different temperature, before the reaction was started. Each measurement was performed in three replicates.

Incubating reactions at different temperatures between 20 and 50 °C showed that a temperature of 32 °C is optimal for VboTA to catalyze reactions (Fig. 3a). Again, with β-homoalanine as substrate the enzyme worked optimally in much more precisely defined reaction conditions. When the reaction temperature was changed by ten degrees from the optimal state, the conversion rate was reduced by 80%. On the other hand, 50% of the activity against β-phenylalanine remained even if the reaction temperature was increased to 50 °C. Measurements of temperature stability revealed that VboTA is heat sensitive with temperatures above 40 °C leading to a rapid inactivation of the enzyme (Fig. 3b).

### 3.5 Determination of Km and V_max_

Four approaches with increasing concentrations (2, 4, 6 and 8 mM) of substrate were conducted, and the amount of the produced glutamate was measured as described in Ref. [47]. The results were plotted in a Lineweaver Burk graph to calculate Km and V_max_. Assuming that VboTA is a homodimer, kcat was also calculated. This assumption is strengthened by the conformation prediction by swiss model [40]. The results are listed in Table 1.

**Table 1:** Kinetic characteristics of VboTA.

	km [mmol l^−1^]	V_max_ [μmol min^−1^]	kcat [s^−1^]	E (kcat/km) [l mmol^−1^ s^−1^]
2-Oxoglutaric acid	2.1 ± 0.4	24.9 ± 2.0	34.2 ± 2.7	16.2 ± 1.3
β-Homophenylalanine	0.7 ± 0.2	1.1 ± 0.05	1.6 ± 0.07	2.2 ± 0.1
β-Leucine	44.7 ± 1.2	48.5 ± 1.2	66.5 ± 7.1	1.5 ± 0.2
β-Homoalanine	4.4 ± 0.3	1.4 ± 0.2	2.0 ± 0.2	0.45 ± 0.05
β-Phenylalanine	24.7 ± 3.8	20.5 ± 2.4	28.1 ± 3.3	1.1 ± 0.1

### 3.6 Substrate specificity

To determine amino acceptor specificity pyruvate, 2-oxoglutarate and oxaloacetate were tested. β-Phenylalanine as an example for aromatic amino acids, β-homoalanine as aliphatic amino acid and γ-amino-pentanoic acid as representative for γ-amino acids were used to test amino donor specificity. Experiments were performed at two pH conditions. The highest activity at pH 8.0 was found for 2-oxoglutarate (0.798 ± 0.023 U μg^−1^ enzyme), which led to an almost three-fold higher activity with β-phenylalanine than pyruvate (0.285 ± 0.005 U μg^−1^ enzyme) or oxaloacetate (0.288 ± 0.004 U μg^−1^ enzyme). On the other hand, the choice of the amino acceptor did not influence the conversion of β-homoalanine at pH 8.0. The only γ-amino acid in this test, γ-amino pentanoic acid, was not accepted as substrate at all.

**Fig. 4:**
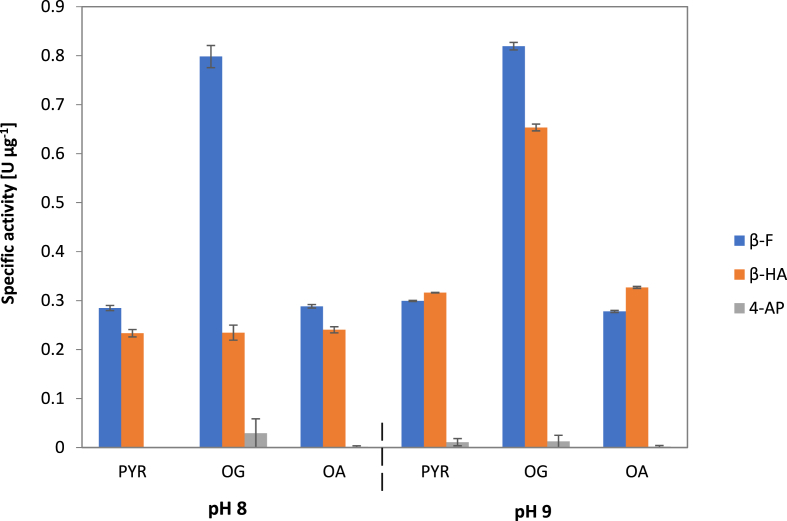
Activity of VboTA, U [μmol min^−1^] after 1 h at 30 °C at pH 8.0 and 9.0 in dependence of β-F or β-HA as amino donors. Each condition was done in two replicates. PYR: pyruvate, OG: 2-oxo-glutarate, OA: oxaloacetate. β-F: β-phenylalanine, β-HA: β-homoalanine, 4-AP: 4-amino-pentanoic acid.

At pH 9.0 there was no significant change of activity compared to pH 8.0, when pyruvate or oxaloacetate were used as amino acceptors. However, with 2-oxoglutarate as amino acceptor, β-phenylalanine conversion remained at a similarly high level (0.819 ± 0.008 U μg^−1^ enzyme). But the conversion of β-homoalanine was increased significantly (p < 0.05) by a factor of almost 3 compared to pH 8.0 (Fig. 4). Because of this higher enzyme activity under more alkaline conditions, the following tests were performed at pH 9.0.

These experiments suggested that only β-amino acids are accepted by VboTA as amino donors.

In order to broaden the substrate spectrum even further, β-alanine, β-homophenylalanine and β-leucine were tested additionally. As representative for branched chain β-amino acids β-leucine, a bulkier aromatic substrate β-homophenylalanine and as a substrate without sidechain β-alanine were used. To confirm the assumption that only β-amino acids are used as amino donors, γ-aminobutyric acid, putrescine, spermine and spermidine were tested for activity, too.

**Fig. 5:**
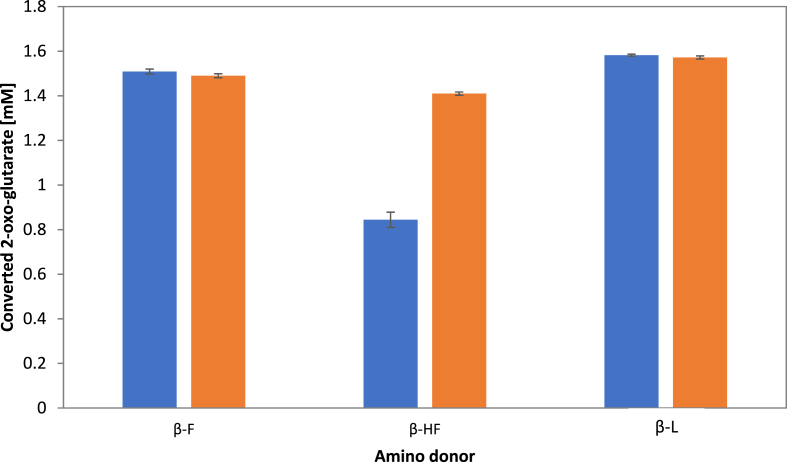
Activity of VboTA, after 1 h at 30 °C at pH 9 (dark grey), and after 3 h at 30 °C at pH 9 (light grey). The amount of converted 2-oxo-glutarate in mM with different amino donors is shown. β-F: β-phenylalanine, β-HF: β-homophenylalanine, β-L: β-leucin. Each measurement was performed in three replicates.

The reaction speed with β-homophenylalanine turned out to be significantly lower than with β-phenylalanine and β-leucine. After 1 h at 30 °C 1.51 mM (or 75.5%) 2-oxo-glutarate were aminated with β-phenylalanine as amino donor and 1.58 mM (or 79.0%) with β-leucine. Using β-homophenylalanine as donor only 0.84 mM (42.0%) could be converted after 1 h. After 3 h the conversion with β-homophenylalanine reached the maximum as the reactions with the other donors. 1.41 mM (70.5%) 2-oxo-glutarate were aminated with β-homophenylalanine, with β-phenylalanine and β-leucine no noteworthy change in amination was observed compared to the level after 1 h (Fig. 5). In the tests with β-alanine, γ-aminobutyric acid, putrescine, spermine and spermidine no activity was observed.

### 3.7 Substrate and product inhibition

To establish if there is an inhibition caused by products or reactants, the activity of VboTA at several concentrations of these compounds was determined (supplemental 5). 2-Oxoglutarate, as the best of the compared amino acceptors, caused strong inhibition at concentrations of more than 10 mM while 20 mM, the highest concentration used, led to a loss of activity of about 70% (supplemental 5). For β-phenylalanine and d-glutamate there was no noteworthy inhibition. In contrast, l-glutamate inhibited VboTA at concentrations above 15 mM, but the effect was not as strong as with 2-oxoglutarate, it caused only a loss of activity of about 20%.

### 3.8 Enzymatic synthesis of β-amino acids by kinetic resolution

To produce enantiopure β-amino acids by VboTA at industrial scale, substrate concentrations higher than 4 mmol L^−1^ have to be deaminated. In our experiments, amino acid concentrations of 50 mmol L^−1^ were used for kinetic resolution and reaction mixtures were analyzed by HPLC after 24 h.

Our assays showed that VboTA deaminated the (R)-enantiomer of β-homophenylalanine and β-homoalanine. In contrast, using β-leucine or β-phenylalanine the (S)-enantiomer was deaminated. Pyruvate and 2-oxoglutarate were used as amino acceptors. In Table 2, the enantiomeric excess (ee) as well as the concentration of the individual amino acid enantiomers after 24 h at 30 °C are listed. Enantiopure (R)-β-leucine, (S)-β-homoalanine and (R)-β-phenylalanine were synthesized with pyruvate as acceptor. If 2-oxoglutarate was used as acceptor, still 5–7 mM of the opposite enantiomer were not deaminated after 24 h, so that ee was between 56.8 ((R)-β-leucine) and 65.6% ((S)-β-homoalanine). For (S)-β-homophenylalanine ee was highest with 2-oxoglutarate (61.6%), whereas 46.7% was found with pyruvate as amino acceptor.

**Table 2:** Concentration and enantiomeric excess (ee) of (S)-β-homophenylalanine, (R)-β-leucine, (S)-β-homoalanine and (R)-β-phenylalanine after kinetic resolution of 50 mM racemic mixtures for 24 h at 30 °C with amino acceptors pyruvate and 2-oxoglutarate.

	(S)-β-homophenylalanine		(R)-β-leucine		(S)-β-homoalanine		(R)-β-phenylalanine	
	ee [%]	c [mM]	ee [%]	c [mM]	ee [%]	c [mM]	ee [%]	c [mM]
Pyruvate	46.7 ± 0.9	25.7 ± 0.1	≥99.9	26.2 ± 0.2	≥99.9	24.8 ± 0.7	≥99.9	25.6 ± 0.6
2-Oxo-glutarate	61.6 ± 1.4	25.5 ± 0.2	56.8 ± 0.2	25.7 ± 0.2	65.6 ± 0.5	25.1 ± 0.0	62.1 ± 2.4	25.8 ± 0.1

The concentration for the enantiomers was still 25 mmol L^−1^, so no deamination of (R)-β-leucine, (S)-β-homoalanine and (R)-β-phenylalanine was observed. Thus, the enzyme catalyses the deamination of the tested β-amino acids stereospecifically giving 25 mmol L^−1^ enantiopure β-amino acid. Reactions with β-homophenylalanine were slower, requiring a longer incubation time or a higher enzyme concentration to generate pure (S)-β-homophenylalanine.

## 4 Discussion

Here we present with VboTA a versatile TA, which catalyses the deamination of β-amino acids. VboTA has been predicted as class III transaminase (Pfam: Aminotran_3 (PF00202) [50]), which is a subclass of fold type I pyridoxal-dependent enzymes. Fold type I enzymes are members of the aspartate aminotransferase family [51]. Originally, class III transaminases were called subclass II instead of class III [51,52], but class III has become a common name for them throughout literature.

The VboTA protein contains all necessary residues of the suggested motif for aromatic β-amino acid transaminases as described in Ref. [44]. In this potential motif two of the residues, V43 and Y76 (V42 and Y75 in VboTA), bind to the aromatic ring of the substrate. Another residue, R41 (R40 in VboTA) forms an ionic bond with the carboxyl group of the substrate. Furthermore, it is supposed that E75 (E74 in VboTA) supports this arginine residue by bending its side chain in position [44]. While this motif was suggested for aromatic β-amino acid transaminases, our data show that this does not exclude the conversion of aliphatic amino acids, since VboTA deaminates aromatic β-amino acids as well as aliphatic ones.

A multiple sequence alignment revealed that all seven sequences have 29 amino acids in common and that aliphatic ω-TAs differ in 32 conserved positions from the four other ω-TAs in the alignment (compare Section 3.2). These more promiscuous ω-TAs have 50 conserved amino acid positions, which are different in aliphatic ω-TAs. There are more conserved residues in the active centre of the more versatile ω-TAs as in the centre of the aliphatic ω-TAs (compare Fig. 1 + 2). Many of these residues appear in the p-pocket, or close to it, and thus might be involved in binding the substrates side chain. Some residues are non-polar, for example A295, G298 and F300, which could be involved in binding aromatic substrates, but the cave is still big enough for branched chained substrates like β-leucine. Interestingly, there are also some polar residues, like arginine and several asparagine residues in the p-pocket, which could keep polar substrates in position, but the activity with polar substrates was not tested. Compared to the versatile ω-TAs, the active centre of the aliphatic ω-TAs is less conserved. Here the p-pocket seems to form a bigger cave than in the more versatile ω-TAs. There are also less conserved residues.

One aromatic ω-TA was also used for a sequence comparison is from *V. paradoxus*. This enzyme has a high sequence homology to VboTA (86% identities, 92% positives). The minor differences in their sequences, however, are sufficient to allow VboTA to accept a larger substrate spectrum as the *V. paradoxus* enzyme, whose activity against β-homoalanine is only 1% of its activity against β-phenylalanine. Despite the high similarity, both enzymes have a different temperature optimum. The *V. paradoxus* enzyme has an optimum at 55 °C, a temperature which quickly inactivates VboTA. Next to the lower temperature optimum, also a higher ee was reached with VboTA [44]. These differences make VboTA interesting as an enzymatically catalyst for the production of β-amino acids.

ω-Transaminases with an activity against both, aromatic and aliphatic amino acids, appear to be quite rare, most of the ω-TAs mentioned by Rudat et al. in 2012 are able to deaminate either aromatic or aliphatic amino acids [42]. Even when both types of amino acids are accepted, the activity is higher for one of them. In an effort to increase the activity against β-phenylalanine, an ω-TA from *Caulobacter crescentus* has been modified, but even after successful improvement the activity against aliphatic amino acids was still 100-fold higher [53]. Interestingly, the enzyme we describe in this study, VboTA, is able to deaminate aliphatic, aromatic and branched chained amino acids at comparable activity, which is a property that even distinguishes it from ω-TAs from other *Variovorax* species. For example, the activity of the ω-TA of *V. paradoxus* towards β-homoalanine is only 1% when compared to its activity towards β-phenylalanine [44]. In contrast, the activity of VboTA towards β-homoalanine is roughly 80% (0.82 μmol ml^−1^ μg^−1^ enzyme for β-phenylalanine and 0.65 μmol ml^−1^ μg^−1^ for β-homoalanine) of the activity towards β-phenylalanine.

VboTA's optimal reaction temperature of 32 °C is quite low, compared to other transaminases, e.g. the ω-TA from *Thermococcus* sp. CKU-1 showed increasing activity up to 95 °C [54]. Also one ω-TA from *Thermomicrobium roseum* displayed increasing activity up to 80 °C [55]. Both mentioned enzymes exhibited low activity at 30 °C, thus VboTA might be interesting, whenever reactions should take place at lower temperatures for reasons of energy efficiency, or heat sensitive substrates.

The above mentioned ω-TA from *Thermococcus* sp. CKU-1 showed a similar pH optimum as VboTA (*Thermococcus*: 9.5, VboTA: 9.0). For an ω-TA from *Variovorax paradoxus*, a pH optimum of 9 was reported, too [56]. Especially for β-homoalanine a shift from pH 8 to 9 had a high impact on the activity with 2-oxo-glutarate as amino acceptor (Fig. 4). There was only a minor effect observed at the activity of the other donor/acceptor pairs. This might be due to the fact, that 2-oxo-glutarate and β-phenylalanine are among the enzymes favorized substrates, and β-phenylalanine can be converted at a high velocity at pH 8, too. The maximum was reached in both experiments. As β-homoalanine is not the most favorized amino donor, it might be converted slowly at pH 8, even with the best amino acceptor, but it profits from the pH shift and the reaction becomes faster. Pyruvate and oxaloacetate are not the enzymes favorized amino acceptors, thus a higher pH value had no big impact on the activities with these acceptors. Such high values seem to be an exception, as in 2011 Tufvesson et al. listed pH values amongst other reaction conditions for a large number of ω-TAs, that were between 7 and 8.5 for most enzymes [57].

VboTA is highly enantioselective. After 24 h incubation time 25 mmol L^−1^ (R)-β-leucine, (R)-β-phenylalanine and (S)-β-homoalanine with an ee of >99.9% were obtained by kinetic resolution of the enantiomeric mixtures (supplemental 6–9). While the activity of VboTA with 2-oxoglutarate as acceptor was higher in the activity assays, only pyruvate assisted kinetic resolution gave enantiopure products. This could be due to inhibition caused by high concentrations of 2-oxoglutarate. Another reason can be a product inhibition by glutamate, which is produced by the amination of 2-oxoglutarate. Here a substrate as well as product inhibition appears (compare to supplemental 5). Both effects might slow down the reaction with 2-oxoglutarate, so that the reaction with pyruvate is faster at those concentrations.

Pyruvate seems to be the better amino acceptor for kinetic resolution of 50 mM β-amino acids, conferring higher ee values in shorter reaction times. This is advantageous for industrial applications, because pyruvate is much cheaper.

In the course of this study only the kinetic resolution reaction type was investigated. For further applications, it would be important to also apply asymmetric synthesis.

Basically, there are transaminases that can perform both, kinetic resolution and asymmetric synthesis. For example, a transaminase from *Chromobacterium violaceum* is used to produce different amines in both ways [58]. Another example is an enzyme from *Vibrio fluvialis*, which is used in an unmodified form to perform kinetic resolution [59], but it was also modified to enable it for the asymmetric synthesis of an intermediate for the production of imagabalin [60]. Whether VboTA is also able to perform in asymmetric synthesis has to be shown in future studies.

Taken together, we propose VboTA as valuable enzyme for biotechnological purposes because of the variety of accepted substrates and its convenient reaction conditions.

## Author contribution statement

Uwe Wegner, Marion Rauter: Conceived and designed the experiments; Performed the experiments; Analyzed and interpreted the data; Wrote the paper.

Falko Matthes, Mohammad-Reza Hajirezaei: Analyzed and interpreted the data.

Gotthard Kunze, Nicolaus von Wirén: Contributed reagents, materials, analysis tools or data.

## Funding statement

This work was supported by Bundesministerium für Wirtschaft und Energie [ZF4061308AJ7].

Nicolaus von Wirén was supported by Deutsche Forschungsgemeinschaft [491250510].

The open access publishing costs were partially funded by the Deutsche Forschungsgemeinschaft (DFG, German Research Foundation, grant 491250510).

## Data availability statement

Data will be made available on request.

## Declaration of interest’s statement

The authors declare no competing interests.
