# Supplementary material for: A transaminase with β-activity from Variovorax boronicumulans for the production of enantiopure β-amino acids
Source: Heliyon. 2022 Dec 30;9(1):e12729. doi: 10.1016/j.heliyon.2022.e12729 (PMC9850050; doi:10.1016/j.heliyon.2022.e12729)
Supplement: Multimedia component 1 [file mmc1.docx]

Query_10001 1 MSA AKL-[ 1]-----------DL---SHLWMPFTANRQ--F-KANPRLLASAKGMYYTSFDGRQILDGTAGLWCVN 56

Query_10002 1 MQK QRTT[ 3]-----------RELDAAHHLHPFTDTAS--LNQAGARVMTRGEGVYLWDSEGNKIIDGMAGLWCVN 63

Query_10003 1 MNK ---- -------PQSWEARAETYSLYGFTDMPS--LHQRGTVVVTHGEGPYIVDVNGRRYLDANSGLWNMV 60

Query_10004 1 MSS GSRR[ 2]NVYREPGSAAADLFERARRVLPGGNTRTTVYSAPYPPYAARGRGAVIVDADGEERLDFVNNYTALI 75

Query_10005 1 MNE[9]ASDT[11]NTFARDNPVSAGHHERARRSMPGGNTRSILFHRPFPLVIAQGTGSRFQDVDGHAYVNFLGEYTAGL 93

Query_10006 1 MT- ---T[10]QRFTDGNPESLRRFEAQTRYMPGANSRSVLFYAPFPLTIAKGEGASLWDADGHRYTDFIAEYTAGV 79

Query_10007 1 MHS ATGT[10]ARYRARNPGSERLLRKAADVLPAGNTRSVLFYTPFPLYMARGEGCHLWDADGHRYLDALGEFTAGI 83

Query_10001 57 AGHCREEIVSAIASQAGVM[3]PGFQLGHPLAFEAATAVAGLMPqGLDRVFFTNSGSESVDTALKIAL[6]GEAQRTRLI 139

Query_10002 64 VGYGRKDFAEAARRQMEEL[4]TFFKTTHPAVVELSSLLAEVTPaGFDRVFYTNSGSESVDTMIRMVR[6]GKPEKKTLI 147

Query_10003 61 AGFDHKGLIDAAKAQYERF[4]AFFGRMSDQTVMLSEKLVEVSPfDSGRVFYTNSGSEANDTMVKMLW[6]GKPQKRKIL 144

Query_10004 76 HGHADPDINEAVIRQLADG VAFAMPTEHEIALAELLTERVP-SLQQVRFTNSGTEAVMMAIKAAR AYTGRPRIA 148

Query_10005 94 FGHSHPVIRAAVERALAVG LNLSTQTENEALFAEAVCDRFP-SIDLVRFTNSGTEANLMALATAT AITGRKTVL 166

Query_10006 80 YGHSAPEIREAVIEAMQGG INLTGHNLLEGRLAQTICERFP-QVEQLRFTNSGTEANLMALTAAL RFTGRRKIV 152

Query_10007 84 YGHSNPVIRQAIVAALQDG LSLSSHTAREAALAHEIQRRFP-GMALLRFTNSGTEANLMALAAAT AHTGRRKVL 156

Query_10001 140 GRERGYHGVGFGG-isVGGISPNRKTFSGALLPAVDHLPHT---HSL[11]WGAHLADELERIIALHDASTIAAVIVEPM 222

Query_10002 148 GRWNGYHGSTIGG-asLGGMKYMHEQG-DLPIPGMAHIEQP---WWY[10]FGVVAARWLEEKILEIGADKVAAFVGEPI 228

Query_10003 145 TRWNAYHGVTAVS---ASMTGKPYNSVFGLPLPGFVHLTCP---HYW[11]FVARLARELEETIQREGADTIAGFFAEPV 225

Query_10004 149 KFDGCYHGSYDFAevsTQSSGKPGEDGFPVATPYTGGTPQAvldSVV VLPFNDIDGTERLIEQHRDELAAVLIDPN 224

Query_10005 167 AFDGGYHGGLLNF----------ASGHAPTNAPY----------HVV LGVYNDVEGTADLLKRHGHDCAAILVEPM 222

Query_10006 153 VFSGGYHGGVLGF----------GDQPSPTTVPF----------DFL VLPYNDADTARAQIDEHGADIAAILVEPM 208

Query_10007 157 VFNGAYHGGVLSF----------GGGGSPVNVPH----------DFV VAPYNDLDAVRGLVQTHGPQLAAILVEPM 212

Query_10001 223 AGSTGVLVPPKGYLEKLREITARHGILLIFDEVITayGRLGEATAAAYFGVTPDLITMAKGVSNAaVPAGAVAVRREVHD 302

Query_10002 229 QGAGGVIVPPATYWPEIERICRKYDVLLVADEVICgfGRTGEWFGHQHFGFQPDLFTAAKGLSSGyLPIGAVFVGKRVAE 308

Query_10003 226 MGAGGVIPPAKGYFQAILPILRKYDIPVISDEVICgfGRTGNTWGCVTYDFTPDAIISSKNLTAGfFPMGAVILGPELSK 305

Query_10004 225 PRSLGLYPAEPAFLQRLREITRAYGIVLIFDEVIS--LRSDYGGMQSVLGVTPDLTAMGKIIGGG-FPVGAVGGSAEVMS 301

Query_10005 223 LGAGGCVPAERAFLDLLRAEASRCGALLIFDEVMT--SRLSGGGAQEMLGISADLTTLGKYIGGG-MSFGAFGGRRDLME 299

Query_10006 209 QGASGCIPGGREFLQTLRGGATRVGALLVFDEVMT--SRLGPHGLANGLGIRADLTTLGKYIGGG-MSFGAFGGRADVMA 285

Query_10007 213 LGAGGCIPAEPAFLHGLRALADACGALLILDEVMT--SRLSGGGRQALLGLKPDLTTLGKYFGGG-LSFGAFGGRVDVMS 289

Query_10001 303 AIVNGPqGGIEFFHGYTYSAHPLAAAAVLATLDIYRREDLFARA-RKLSAAFEEAAHSLKGA-PHVIDVRNIGLVAGIEL 380

Query_10002 309 GLIAGG----DFNHGFTYSGHPVCAAVAHANVAALRDEGIVQRVkDDIGPYMQKRWRETFSRFEHVDDVRGVGMVQAFTL 384

Query_10003 306 RLETAIeAIEEFPHGFTASGHPVGCAIALKAIDVVMNEGLAENV-RRLAPRFEERLKHIAER-PNIGEYRGIGFMWALEA 383

Query_10004 302 VFDPTG-GPPRAPHGGTFNANPVTMVAGLTAMRKLTPAEF-DRL-ATLGQQLRAGVEEVLREAGVPGQVTGYGSLFHIHL 378

Query_10005 300 RFDPARdG--AFAHAGTFNNNILTMSAGHAALTQIYTRQAASDL-SASGDRFRANLNRIAVENQAPLQFTGLGSLGTIHF 376

Query_10006 286 QFDPRT-G-ALA-HSGTFNNNVMTMAAGYAGLTQLFTPEAAAAL-AERGEAMRSRLNALCEREGVAMQFTGVGSLMNAHF 361

Query_10007 290 RFDPRR-ADALG-HAGTFNNNTLTMAAGLAGLTQVLTPAALDAL-NQRGERLRERLNGVFKRHAVGLQFTGLGSVMQLHA 366

Query_10001 381 SPREGAPG------ARAAEAFQKCFDT----GLMVRYTGDILAVSPpliVDENQIGQIFE----GIGKVLKEVA----- 440

Query_10002 385 VKNKAKRELFpdF----GEIGTLCRDIFFRNNLIMRACGDHIVSAPplvMTRAEVDEMLAVAERCLEEFEQTLKARGla 459

Query_10003 384 VKDKASKTPFdgNLSVSERIANTCTDL----GLICRPLGQSVVLCPpfiLTEAQMDEMFDKLEKALDKVFAEVAAAAle 458

Query_10004 379 HQRPLADYRNsvLSAQERAFVGRVHEALMGRGIFITPALFGCLSTP---MGVPEVEAFVDAFAAALQDARG-------- 446

Query_10005 377 SRAPIRSAGD--VRAADQQLKELFFFHMLRKGIYLAPRGMYALSLE---IADAGRDAFAEALADFIGEQRALLM----- 445

Query_10006 362 LRGEVRRVAD--LAAVDGRLRQLLFFHLLKEGIYTSPRGFVVLSLP---LSAQDIDRYVAAVGSFIEACRPLLLSAG-- 433

Query_10007 367 TDRPLRNAAD--LAGADDRVKALLFFDLLERGIFLARRGLVALSLP---FGDAEADEFVAALDAVVTARHALLPVAG-- 438

|  lcl\|Query_10001 | AAP92672.1 omega-amino acid:pyruvate transaminase [*Achromobacter denitrificans*] |  |
| --- | --- | --- |
|  lcl\|Query_10002 | WP_011135573.1 aspartate aminotransferase family protein [*Chromobacterium violaceum*] |  |
|  lcl\|Query_10003 | pdb\|3NUI\|A Chain A, Pyruvate transaminase |  |
|  lcl\|Query_10004 | WP_012871332.1 aspartate aminotransferase family protein [*Sphaerobacter thermophilus*] |  |
|  lcl\|Query_10005 | ABL74379.1 beta-transaminase [*Mesorhizobium* sp. LUK] |  |
|  lcl\|Query_10006 | WP_095950167.1 aminotransferase class III-fold pyridoxal phosphate-dependent enzyme [*Variovorax boronicumulans*] |  |
|  lcl\|Query_10007 | WP_041388512.1 aminotransferase class III-fold pyridoxal phosphate-dependent enzyme [*Polaromonas* sp. JS666] |  |

*Supplemental 1: Sequence alignment of different ω-TAs. The first three enzymes accept only aliphatic amino acids as substrate, the last four accept aliphatic as well as aromatic amino acids. In red: residues which are conserved in all seven sequences; in grey: gaps between the sequences; in blue: all other residues.*


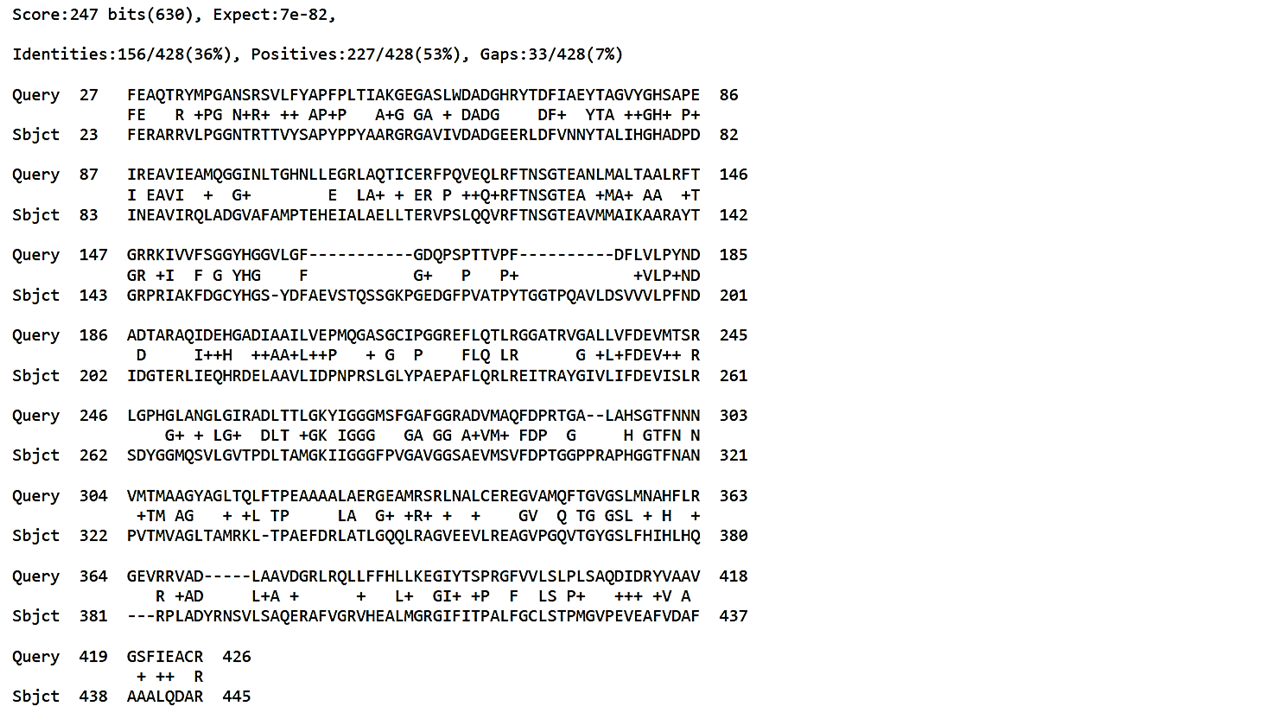


Supplemental 2: Blast result VboTA vs StoTA. Query: sequence of VboTA (NCBI accession WP_095950167.1). Sbjct: sequence of StoTA (NCBI accession WP_012871332.1). Method: Compositional matrix adjust. The number of 53 % positive residues imply, that VboTA might have a similar function like StoTA.

**
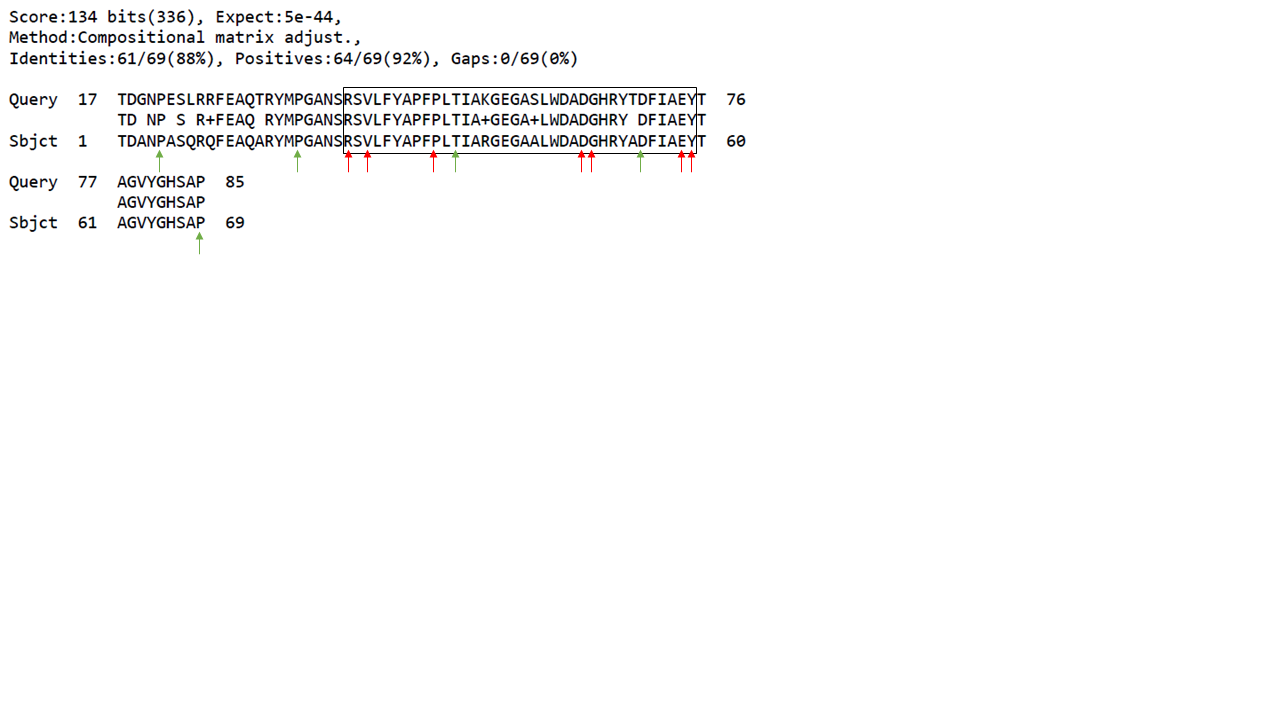
**

Supplemental 3: Blast of VboTA against a potential motif of ω-transaminases [44]. Query: sequence of motif. Sbjct: part of VboTA sequence. The potential functional domain of the motif is in the box. Green arrows mark borders of secondary structures, red arrows mark conserved residues, which seem to be important for the function of aromatic β-transaminases [44].


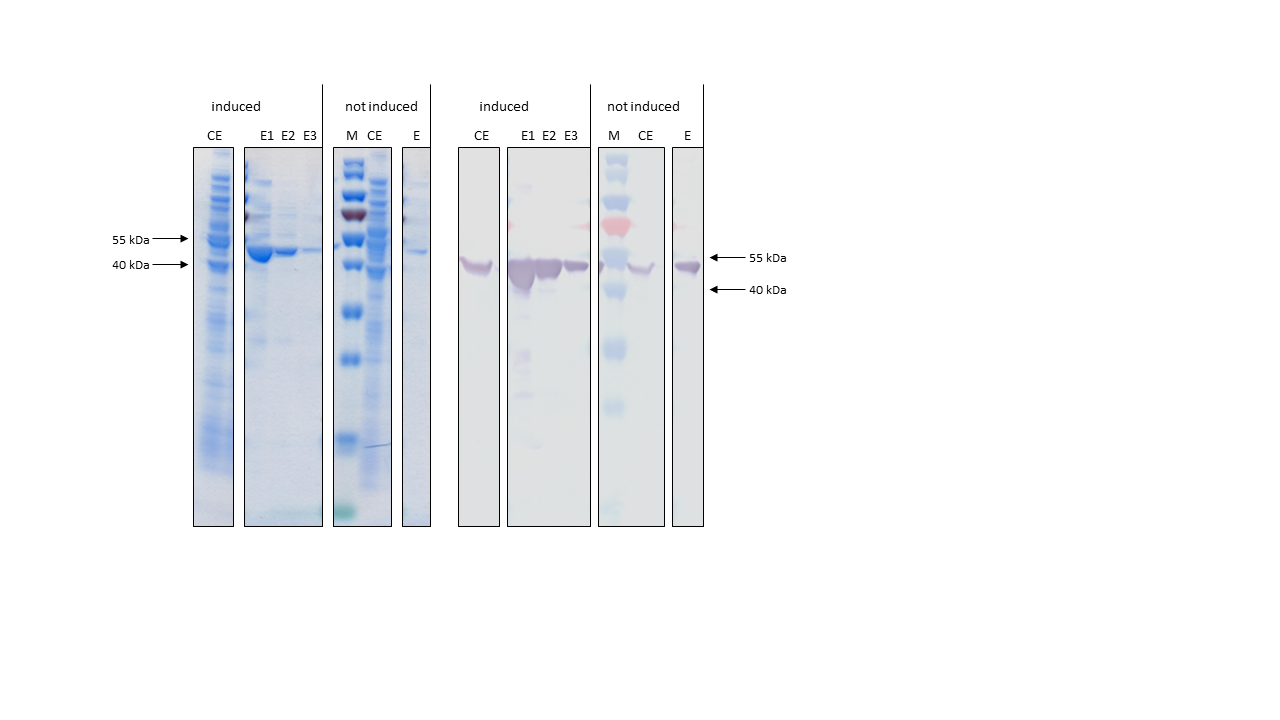


Supplemental 4: Purification of VboTA. Left: Coomassie-stained SDS-PAGE. Right: Immunostained Western blot with anti-His antibody. CE: crude extract, E: eluate. Calculated molecular mass of VboTA (including His-Tag): 47.17 kDa. Washing steps are not shown.

Supplemental 5: Substrate/product inhibition. The activity of VboTA at pH 9 and at 30 °C, and at the presence of different concentrations of L-glutamate, D-glutamate, 2-oxo-glutarate and β-phenylalanine was determined. The highest activity of each approach was set to 100 %, the other values were calculated accordingly. Each measurement was done in triplicates.

**b)**

**a)**

*Supplemental 6: Separation of β-homoalanine by HPLC-UV.*

*RT((S)-β-homoalanine) = 46.3 min, RT((R)-β-homoalanine) = 56.5 min*

1. *Control of the kinetic resolution of β-homoalanine with pyruvate as amino acceptor. Instead of enzyme water was added to the reaction mixture.*
2. *Kinetic resolution of β-homoalanine by VboTA with pyruvate as amino acceptor as described in 2.11 after 24 h of incubation.*

**b)**

**a)**

*Supplemental 7: Separation of β-leucine by HPLC-UV.*

*RT((R)-β-leucine) = 5.6 min, RT((S)-β-leucine) = 6.6 min*

1. *Control of the kinetic resolution of β-leucine with pyruvate as amino acceptor. Instead of enzyme water was added to the reaction mixture.*
2. *Kinetic resolution of β-leucine by VboTA with pyruvate as amino acceptor as described in 2.11 after 24 h of incubation.*

**b)**

**a)**

*Supplemental 8: Separation of β- phenylalanine by HPLC-UV.*

*RT((R)-β-phenylalanine) = 9.8 min, RT((S)-β- phenylalanine) = 11.7 min*

1. *Control of the kinetic resolution of β- phenylalanine with pyruvate as amino acceptor. Instead of enzyme water was added to the reaction mixture.*
2. *Kinetic resolution of β- phenylalanine by VboTA with pyruvate as amino acceptor as described in in 2.11 after 24 h of incubation.*

**b)**

**a)**

*Supplemental 9: Separation of β-homophenylalanine by HPLC-UV.*

*RT((S)-β-homophenylalanine) = 10.5 min, RT((R)-β-homophenylalanine) = 12.4 min*

1. *Control of the kinetic resolution of β-homophenylalanine with pyruvate as amino acceptor. Instead of enzyme water was added to the reaction mixture.*
2. *Kinetic resolution of β-homophenylalanine by VboTA with pyruvate as amino acceptor as described in in 2.11 after 24 h of incubation.*
